# Supplementary material for: Lactobacillus gasseri ATCC33323 affects the intestinal mucosal barrier to ameliorate DSS-induced colitis through the NR1I3-mediated regulation of E-cadherin
Source: PLoS Pathog. 2024 Sep 9;20(9):e1012541. doi: 10.1371/journal.ppat.1012541 (PMC11412683; doi:10.1371/journal.ppat.1012541)
Supplement: S1 Table — (DOCX) [file ppat.1012541.s001.docx]

**SUPPLEMENTARY TABLES**

**Supplemental Table 1:** Sequences of the primers used for real-time reverse transcription PCR.

| Gene | Primer sequence （5‘→3‘） |
| --- | --- |
| IL-1β-mouse | F: AATCTCGCAGCAGCACATCAAC |
|  | R: AGGTCCACGGGAAAGACACAG |
| IL-6-mouse | F: GAGAGGAGACTTCACAGAGGATACC |
|  | R: TCATTTCCACGATTTCCCAGAGAAC |
| TNFα-mouse | F: CACGCTCTTCTGTCTACTGAACTTC |
|  | R: CTTGGTGGTTTGTGAGTGTGAGG |
| β-actin-mouse | F: TGCTGTCCCTGTATGCCTCTGG |
|  | R: ACCGCTCGTTGCCAATAGTGATG |
| IL-1β-human | F: ATGGCTTATTACAGTGGCAATGAGG |
|  | R: AGTGGTGGTCGGAGATTCGTAG |
| IL-6-human | F: TTCGGTCCAGTTGCCTTCTCC |
|  | R: TCTGAAGAGGTGAGTGGCTGTC |
| TNFα-human | F: TCAACCTCCTCTCTGCCATCAAG |
|  | R: CGCTGAGTCGGTCACCCTTC |
| GAPDH-human | F: CACCCACTCCTCCACCTTTGAC |
|  | R: GTCCACCACCCTGTTGCTGTAG |
| CDH1 | F: CTGCTGCTCTTGCTGTTTCTTCG |
|  | R: CTCTTCTCCGCCTCCTTCTTCATC |
| IGF2 | F: CCGTGGCATCGTTGAGGAGTG |
|  | R: CGGGGTATCTGGGGAAGTTGTC |
| PROX1 | F: CCTGAGCCACCACCCTTGTTC |
|  | R: TCTTGAAGATCGCCGCACTCG |
| ATP2A1 | F: TGGTGCTGGCTGACGACAAC |
|  | R: TTGGAGGAAATGAGGTAGCGGATG |
| H2-OB | F: ACAACCTGCTGCTCTGCTCTG |
|  | R: AGACCTCTCCTCCTGTCCATTCC |
| ESCO2 | F: ATTCCAATGCTCCTCGGGTTCTG |
|  | R: TCCCAGTGACGGCTCATTTTCC |
| MKI67 | F: TCAAGCCACAGTCCAAGAGAAGTC |
|  | R: TCCCTACTGATGGTGTTCGTTTCC |
| LRR1 | F: GAGCCGTGTTGAGCCTCTGTC |
|  | R: AGGGTGGAGATGAGCAGGAAGG |
| SYT8 | F: CTCCTCGTCTCCTGCCTCCTC |
|  | R: CTTGTCCCTGGGCTTCTTCCTG |
| BIRC5 | F: AAGGACCACCGCATCTCTACATTC |
|  | R: CTCGTTCTCAGTGGGGCAGTG |
| PLA2G4E | F: CCAGGAGGCGGAGGAAGAGG |
|  | R: GAGGTTGGGCAGCAGGGTTC |
| UHRF1 | F: GAAGCGGAAGTCGGCAGGAG |
|  | R: TTGGCGTTGCTCTTGTCCTCTC |
| EPHB3 | F: TACGGCTCAATGACGGACAGTTC |
|  | R: AGGATGTTTCGGGCAGCAAGG |
| NR1I3 | F: CACCTGGAACACCCTGGATACAC |
|  | R: AACCACTGGGCTCCCTTTGAAC |
| PBK | F: ACTGCTCCTGCCTTCATAACCATC |
|  | R: CTGTGCCAATGTAACAAGCCTCAG |
| GAPDH | F: CACCCACTCCTCCACCTTTGAC |
|  | R: GTCCACCACCCTGTTGCTGTAG |

F=forward; R=reverse
