# Supplementary figures and images for: Lactobacillus gasseri ATCC33323 affects the intestinal mucosal barrier to ameliorate DSS-induced colitis through the NR1I3-mediated regulation of E-cadherin
Source: PLoS Pathog. 2024 Sep 9;20(9):e1012541. doi: 10.1371/journal.ppat.1012541 (PMC11412683; doi:10.1371/journal.ppat.1012541)

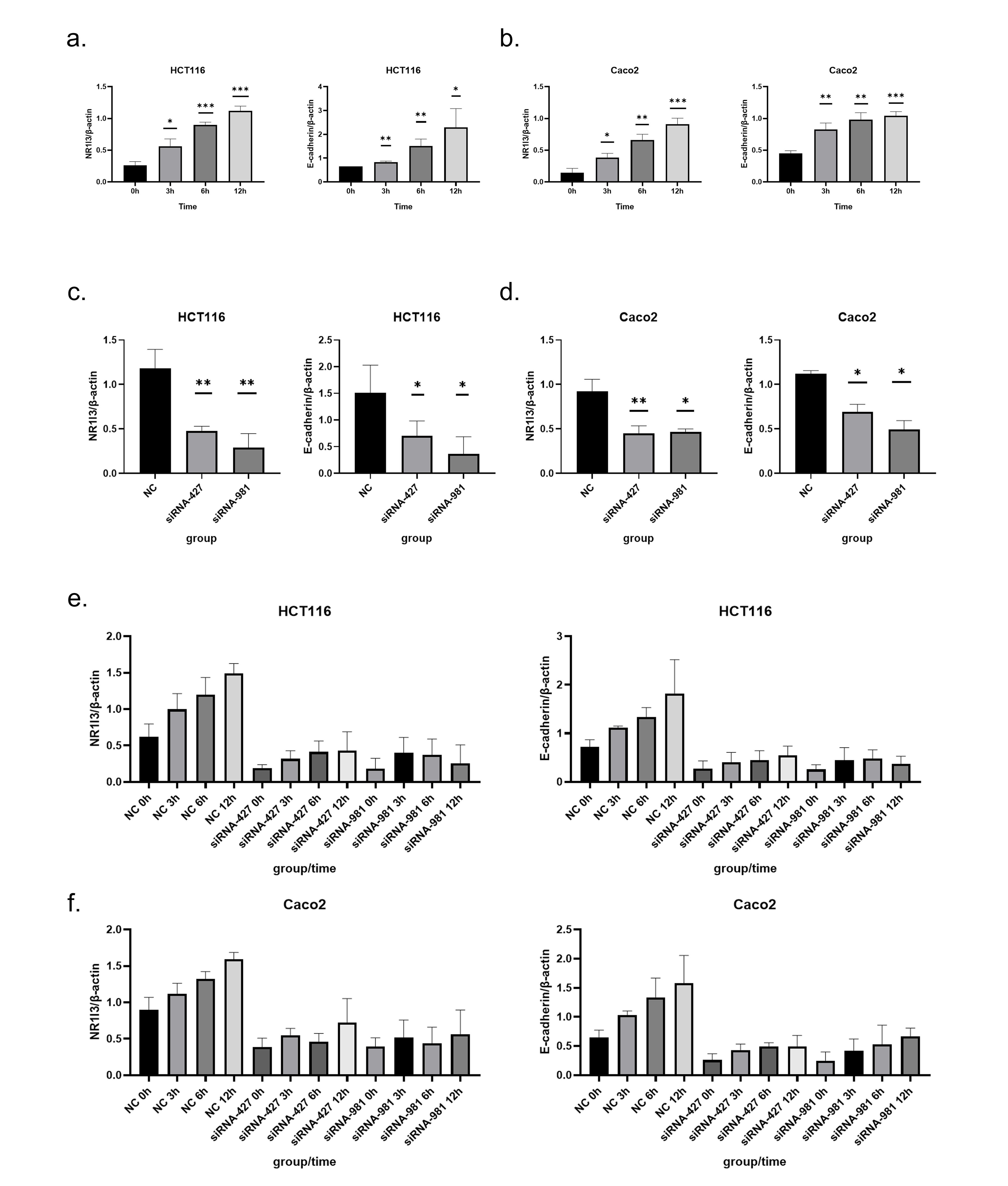

Supplement: S1 Fig — a. Analysis of E-cadherin and NR1I3 grayscale values in the HCT116 cell line in Fig 7B; b. Analysis of E-cadherin and NR1I3 grayscale values in the Caco2 cell line in Fig 7B; c. Analysis of E-cadherin and NR1I3 grayscale values in the HCT116 cell line in Fig 7C; d. Analysis of E-cadherin and NR1I3 grayscale values in the Caco2 cell line in Fig 7C; e. Analysis of E-cadherin and NR1I3 grayscale values in the HCT116 cell line in Fig 7D; f. Analysis of E-cadherin and NR1I3 grayscale values in the Caco2 cell line in Fig 7D. The values are expressed as the means ± SDs (n = 3). Superscript letters indicate significant differences at *P < 0.05, **P < 0.01, and ***P < 0.001. (TIF) [file ppat.1012541.s002.tif]

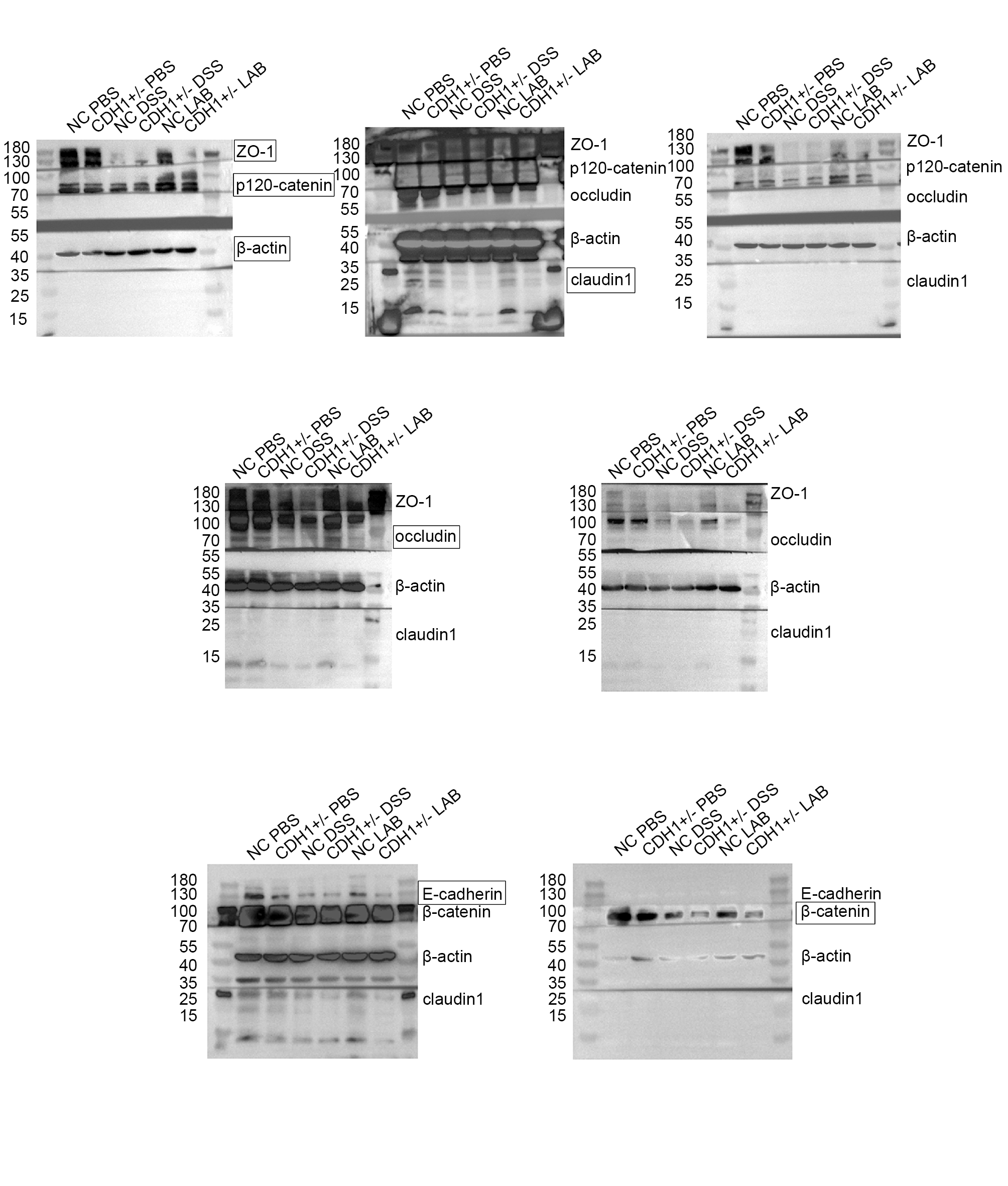

Supplement: S2 Fig — (TIF) [file ppat.1012541.s003.tif]

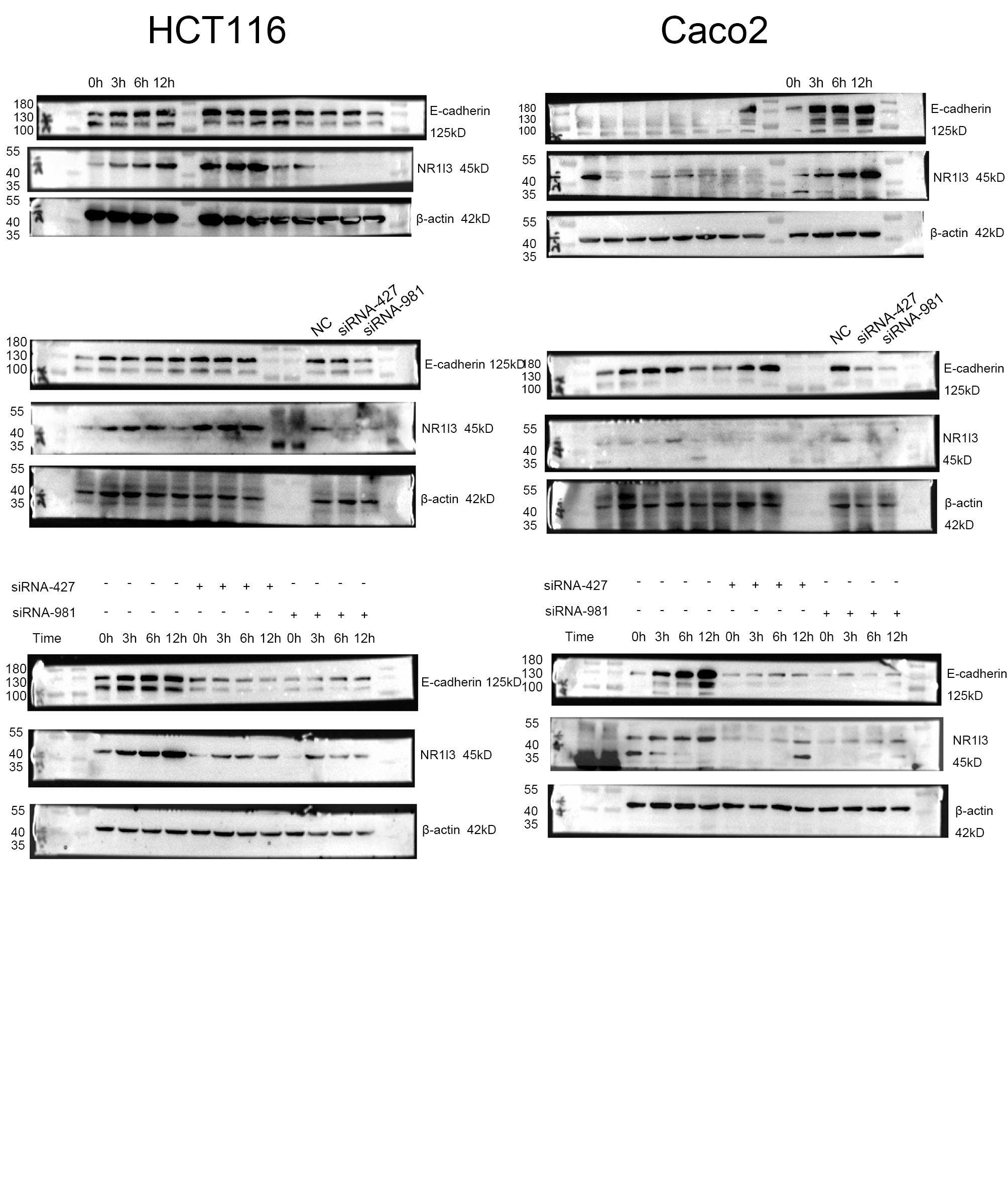

Supplement: S3 Fig — (TIF) [file ppat.1012541.s004.tif]
