# Supplementary material for: Lactobacillus gasseri ATCC33323 affects the intestinal mucosal barrier to ameliorate DSS-induced colitis through the NR1I3-mediated regulation of E-cadherin
Source: PLoS Pathog. 2024 Sep 9;20(9):e1012541. doi: 10.1371/journal.ppat.1012541 (PMC11412683; doi:10.1371/journal.ppat.1012541)
Supplement: Attachment — Submitted filename: response letter.docx [file ppat.1012541.s005.docx]

Dear editors and reviewers,

We are very grateful for your constructive comments and suggestions for our manuscript entitled "*Lactobacillus gasseri* ATCC33323 affects the intestinal mucosal barrier to ameliorate DSS-induced colitis through the NR1I3-mediated regulation of E-cadherin" (PPATHOGENS-D-24-00336). Your comments were very valuable and helpful for improving our manuscript. In the following, our responses to all of the comments are provided individually.

We have tried our best to make all of the revisions clear, and we hope that the revised manuscript satisfies the requirements for publication. In accordance with the comments of the associate editor and reviewers, we have extensively modified the manuscript and supplemented it with additional data to ensure that our results are convincing. The reviewers’ comments are listed below in italicized font, and each specific concern has been addressed. Our responses are in normal font, and changes/additions to the manuscript are in the blue text. Thank you again for your positive comments and valuable suggestions to improve the quality of our manuscript.

Thank you for taking the time to consider our research, and we look forward to hearing from you at your earliest convenience.

Yours sincerely,

Jianping Zhou

Corresponding Author

The First Hospital of China Medical University

Replies to each reviewer’s comments

Reviewer #1

***1. Lines 383-84- the authors mentioned a difference in mortality rate between the groups. Please replace Figure 1d, whose normalise data is presented in Figure 1e, with a survival curve to support the statement.***

Response: We sincerely thank the reviewer for their careful reading. As suggested by the reviewer, we have replaced Figure 1d with a survival curve (Lines 416, 1218).

***2. Lines 393-95 please revise this statement as it doesn’t agree with the data in Figure 1f and the text in lines 395-98 “Similarly, the improvement in colon length was significantly reduced, and intestinal wall congestion was visible in the CDH1+/- LAB group”.***

Response: Thank you for your insightful comment and kind suggestion. We have rewritten this part according to the reviewer’s suggestion. Lines 423-425 "In the NC LAB group, there was no significant shortening of colon length, and no congestion or hematoma was observed."

***3. Figure 4, the immunofluorescent stainings of b-catenin and p120-catenin are difficult to see and even more when they’re merged. Higher zoom might be helpful.***

Response: Thank you for your insightful question. We think this is an excellent suggestion. We have redone the immunofluorescence experiment, selected a 400× microscope field of view for plotting and statistical analysis on the basis of the reviewer's comment, and modified Figure 4 to make the immunofluorescence staining of b-catenin and p120-catenin more visual (Lines 1238-1240).

***4. Line 705-706 – the authors state “…E-cadherin semiknockout mice exhibit accelerated progression of colitis, which indicates that E-cadherin plays a crucial role in intestinal protection…”, which is not really in line with the data presented in this manuscript.***

Response: Thank you for your kind suggestion. Considering the reviewer’s comment, we have deleted “...E-cadherin semiknockout mice exhibit accelerated progression of colitis, which indicates that E-cadherin plays a crucial role in intestinal protection.”

***5. Line 68-69 the authors state “…on probiotics, which have shown strong efficacy in the treatment of IBD[2]”. To date, the use of probiotics in IBD hasn’t been as successful as seen in preclinical models. Some efficacy studies have been reported in UC but not in CD. Please revise this strong statement and adapt according to current literature.***

Response: Thank you for your insightful suggestion. We have modified the sentence according to the reviewer’s comment. We have replaced “Therefore, people have begun to focus on probiotics, which have shown strong efficacy in the treatment of IBD[2],” with “Therefore, researchers have begun to focus on probiotics, which have shown strong efficacy in mouse models of intestinal inflammation and have been found to influence clinical effects in patients with UC[2, 3].” and modified the literature citations here (Lines 49-52).

***6. Line 81 – correct “zona encludens” with “zona occludens”***

Response: We sincerely thank the reviewer for their careful reading. As suggested by the reviewer, we have corrected “zona encludens” to “zona occludens” (Line 64).

***7. Line 89-90 – please revise the sentence “… these strains include ZO-1, occludin, claudin1, β-catenin, and E-cadherin”. I believe the authors are referring to junction proteins regulated by different Lactobacilli strains.***

Response: Thank you for your patience and friendly reminder. We apologize for this careless mistake. The relevant sentences have been modified to “as several strains of the Lactobacillus genus have been shown to regulate cytokine activity, improve the integrity of the intestinal barrier, and affect proteins such as ZO-1, occludin, claudin1, β-catenin, and E-cadherin.” (Lines 70-73).

***8. Lines 103-05 – what is the relevance of the 70 possible transcription factors identified in L. gasseri in context of this study or IBD? Please clarify or delete the sentence.***

Response: Thank you very much for the constructive feedback. As suggested by the reviewer, we have deleted this sentence.

***9. NR1I3 is first time mentioned in line 180, introduce this factor in the introduction and why is relevant for the studies***

Response: We sincerely appreciate this valuable comment. We have added “The constitutive androstane receptor (CAR, NR1I3) is a member of the nuclear receptor superfamily. NR1I3 is a well-known transcription factor that can regulate the expression of many genes involved in the metabolism and transportation of exogenous and endogenous chemicals[17]. The gut microbiota can affect the expression of NR1I3 target genes, the receptors themselves, and the activity of NR1I3[18].” We describe why NR1I3 is relevant to the experiment in Lines 99-103.

***10. Line 134 & 136 - please clarify if the group labelled as CDH1+/- LAB and NC LAB groups, are also exposed to DSS.***

Response: We apologize that this part was not clear in the original manuscript. Considering the reviewer’s suggestion, we have revised the contents of this section. We have moved the section on the treatment conditions according to exposure to DSS up to the beginning of the mouse grouping so that readers can easily visualize our intervention factors (Lines 128-135).

***11. Please add references to the methodology section, where appropriate.***

Response: We thank the reviewer for reminding us of this important point. We have added the related references (19/20/21/22/23/24) to the revised manuscript (Lines 191, 205, 230, 316, 322, 324).

***12. Line 210-11 …”the supernatant was collected from the eyeball blood”… please revise if you mean plasma or serum was collected.***

Response: Thank you for your insightful question. We apologize for our incorrect description. We have changed this sentence to “...and the serum was collected after 4 hours.” (Lines 216-217).

***13. Line 222 – information on the microscope is missing***

Response: We thank the reviewer for this highly valuable comment. We have added “...microscope (Nikon E100, Japan)” to line 227.

***14. Line 275 – which IL1 expression was assay e.g. IL1b?***

Response: We apologize for our careless mistake. As suggested by the reviewer, we have corrected “IL-1” to “IL-1β” in the resubmitted manuscript (Line 280).

***15. Line 286 – what is the abbreviation CTAB?***

Response: In response to the reviewer’s comment, comprehensive descriptions of abbreviations have been added to the revised manuscript. For instance, “hexadecyl trimethyl ammonium bromide (CTAB)” in Line 291.

***16. Figure 1 c,d,e – please utilise the same colour for the same group to easily visualise the data for the reader***

Response: Thank you for pointing out this problem. To make it easier for the reader to visualize the data, we have unified the colors in Figures 1c, d, and e.

***17. Figure 1b – provide ID on top of the 2 gels for clarity***

Response: Thank you very much for the strong support of our work. To improve the clarity, we have recreated Figure 1b and labeled it with the ID at the top.

***18. Lines 401-03*** ***revise this statement as no significant difference was observed between NC DSS and CDH1+/- DSS to their respective controls “The results illustrated that the diffusion of FITC-dextran in the intestine of mice was significantly increased after supplementation with DSS…”***

Response: Thank you for your insightful question. The relevant sentences have been modified as follows: “The results revealed that the lowest intestinal permeability of the mice was observed in the NC PBS and CDH1+/- PBS groups, and the intestinal permeability of the mice in the NC LAB group was lower than that of the mice in the NC DSS group (P<0.05) and the CDH1+/- LAB group (P<0.001), whereas there was no significant difference in the intestinal permeability of the mice in the CDH1+/- LAB group compared with that of the CDH1+/- DSS group” (Lines 433-439).

***19. Figure 2f, the PCR values are negative in the less inflamed and control animals, can you comment as to what was used as calibrator group in the calculations and include in the legend what the dotted line at zero stands for.***

Response: Thank you very much for the positive feedback and constructive points. We apologize for our incorrect description. For the qPCR results section, we re-performed the statistical analysis and normalized the data by performing a fold change calculation with 2^-ΔΔCT^ values. Moreover, we have redrawn Figure 2f to visualize the results of the experiment.

***20. Refer in the text where appropriate to suppl figures 1 and 2 regarding the analysis of WB and staining of the representative figures 2-4.***

Response: We thank the reviewer for bringing this to our attention. We have placed the WB analysis plots in Figure 2h, the staining analysis results in Figure 3f, and the staining analysis results in Figures 4b&d (Lines 1229, 1235-1237, 1241-1245).

Reviewer #2

***1. The results indicate that Lactobacillus gasseri ATCC33323 can improve physiological damage in mice with colitis, but why does the title of result 2.1 (P357) suggest that Lactobacillus gasseri ATCC33323 causes physiological damage in DSS mice?***

Response: We thank the reviewer for giving us the opportunity to clarify this point, and apologize for our carelessness. We have corrected the title of the Results section 2.1 to read "E-cadherin is involved in the attenuation of DSS-induced physiological lesions in mice by *L. gasseri* ATCC33323" (Lines 387-388).

***2. Why do results 2.1 and 2.2 have identical titles? Additionally, several titles do not adequately reflect the conclusions drawn.***

Response: We thank the reviewer for pointing out this potential confounding factor. In accordance with your comment, we have modified the titles of some of the results, including in Section 2.2 “E-cadherin is participating in the alleviation of DSS-induced pathological lesions and intestinal permeability changes in mice by *L. gasseri* ATCC33323”, Section 2.3 “E-cadherin is involved in the anti-inflammatory effects of *L. gasseri* ATCC33323 in colitis”, Section 2.4 “*L. gasseri* ATCC33323 affects junction protein expression through E-cadherin in colitis”, and Section 2.5 “Role of E-cadherin in the effects of *L. gasseri* ATCC33323 on gut microbes in mice with colitis” (Lines 417-419, 462-463, 491-492, 538-539).

***3. What was the basis for selecting the following genes (IGF2, ATP2A1, PROX1, H2-OB, ESCO2, MKI67, LRR1, SYT8, BIRC5, PLA2G4E, UHRF1, EPHB3, NR1I3, and PBK), and subsequently, why was NR1I3 singled out? Given the results in Figure 7a-b, the change in NR1I3 is minimal, and the absence of statistical analysis of qPCR results, along with histograms lacking bars, calls the experimental conclusions into question.***

Response: We are grateful for this suggestion. To be clearer and in accordance with the reviewer’s concern, we have added a brief description of the rationale for selecting these genes (Lines 627-632, “On the basis of the differential genes in the RNA-seq results, we selected ESCO2, MKI67, SYT8, BIRC5, PLA2G4E, UHRF1, and NR1I3 among the top 25 genes in terms of the q-values, as well as other meaningful differential genes that have been reported in the literature to be related to inflammation or the intestines, IGF2, ATP2A1, PROX1, H2-OB, LRR1, EPHB3, and PBK[31-37].”). We chose the NR1I3 gene because NR1I3 expression after cell intervention with *L. gasseri* ATCC33323 in an in vitro study was consistent with the results of RNA-seq, and NR1I3 was found to be identical to the expression trend of the CDH1 gene. In the revised manuscript, the corresponding explanation and conclusion have been added, which can be found in lines 634-637 and are given below: “The expression of the CDH1 gene in colon cancer cells increased with prolonged intervention with *L. gasseri* ATCC33323. Similarly, according to the qPCR results, NR1I3 expression also increased continuously in response to *L. gasseri* ATCC33323, which was consistent with the RNA-seq results”. We supplemented the data in the qPCR results in Fig. 7a-b with histograms and analyzed the results statistically to supplement the information for a clearer and more intuitive presentation of our experimental results.

***4. The authors did not analyze fecal DNA from each mouse group to determine the content of Lactobacillus gasseri ATCC33323, failing to demonstrate the efficacy of the microbial transplant. The 16s RNA sequencing results also do not reflect an increase in lactic acid bacteria abundance.***

Response: We thank the reviewer for providing valuable feedback. In accordance with the reviewer’s comment, we have added new analyses to further strengthen our work. Since 16SrDNA analysis allows for only relative quantification of microorganisms, we designed plasmids to generate standard curves and perform qPCR experiments to determine the copy numbers of *Lactobacillus* spp. and *L. gasseri* ATCC33323 in the DNA of each group of mouse fecal samples. We also analyzed the microorganisms for absolute quantification to determine the content of *Lactobacillus* spp. and *L. gasseri* ATCC33323 in the feces of each group of mice and to prove the effectiveness of microbial transplantation. The related experiments were added to the Materials and Methods, Results, and Discussion sections and Figure 5h&i (Lines 308-333, 580-596, 822-831, 1255-1260).

***5. Please explain the rationale behind using colon cancer cells for in vivo experiments. Why not stimulate normal colon cells with pro-inflammatory factors to simulate the in vivo environment?***

Response: We sincerely appreciate these significant suggestions. To address the concern of the reviewer, we performed additional experiments in which colon cancer cells were stimulated with LPS to make our findings more convincing. We stimulated HCT116 and Caco2 colon cancer cell lines with LPS to mimic the inflammatory environment in vitro and observed changes in E-cadherin expression and inflammatory factors after pretreatment with L. gasseri ATCC33323. The related experiments were added to the Materials and Methods, Results, Discussion, and Conclusion sections and Figure 8. We have added the primer sequences in Supplementary Fig. 1 (Lines 180-192, 686-705, 898-905, 922-924, 1281-1290).

Thank you again for your suggestion. We fully understand your question related to our choice of cell lines. Yoshio Araki et al. reported that the human colon cancer-derived Caco2 intestinal epithelial cell system has been widely used as an in vitro model of the intestinal epithelium (“In vitro effects of dextran sulfate sodium on a Caco-2 cell line and plausible mechanisms for dextran sulfate sodium-induced colitis”, 2006). Therefore, we used Caco2 cells as a monolayer model of the intestinal epithelium. It has been demonstrated in the literature that Caco2 can be used as a cell line for in vitro studies of intestinal barrier function ([Min Yu](https://pubmed.ncbi.nlm.nih.gov/?term=Yu+M&cauthor_id=29483826) et al., “Aryl Hydrocarbon Receptor Activation Modulates Intestinal Epithelial Barrier Function by Maintaining Tight Junction Integrity”, 2018, and [Jixiang Zhang](https://pubmed.ncbi.nlm.nih.gov/?term=Zhang+J&cauthor_id=32035144) et al., “Hesperetin ameliorates DSS-induced colitis by maintaining the epithelial barrier via blocking RIPK3/MLKL necroptosis signaling”, 2020). The HCT116 cell line was chosen primarily because of the extensive literature reporting that this cell line is commonly used for in vitro studies of UC ([Jie Zhang](https://pubmed.ncbi.nlm.nih.gov/?term=Zhang+J&cauthor_id=32075910) et al., “Deficiency in the anti-apoptotic protein DJ-1 promotes intestinal epithelial cell apoptosis and aggravates inflammatory bowel disease via p53”, 2020, [Na Sheng](https://pubmed.ncbi.nlm.nih.gov/?term=Sheng+N&cauthor_id=32859970) et al., “Cholesterol 25-hydroxylase protects against experimental colitis in mice by modulating epithelial gut barrier function”, 2020, and [Shuo Gao](https://pubmed.ncbi.nlm.nih.gov/?term=Gao+S&cauthor_id=38004239) et al., “Vitamin D Attenuates Ulcerative Colitis by Inhibiting ACSL4-Mediated Ferroptosis”, 2023).

***6. The western blots fail to display the molecular weight of each protein, casting doubt on the experimental conclusions.***

Response: We apologize that this part was not clear in the original manuscript. Considering the reviewer’s suggestion, we have labeled the molecular weight of each protein in all western blots on the right side of the image (Figures 2, 7, 8).

Reviewer #3

***While there is a lot of work in this study, it is not possible to interpret the data and their potential significance because of the study design. Indeed, based on the description of the study design, the two starting mouse lines are Cdh1flox/wt from a Cdh1flox/flox x Villin-Cre cross versus some control Cdh1 wt/wt mice (where were those mice coming from?). To allow interpretation, the control group should have been littermate mice of the Cdh1flox/flox group. In the absence of controlling for littermates, no conclusion can be reached because of the confounding effect of the microbiota.***

Response: We apologize for confusing the reviewer with a description of the genotype of the control mice. In the original manuscript, the context of Cdh1 WT mice actually referred to Cdh1^wt/flox^ mice. The Cdh1^wt/flox^ mice were derived from a cross between mice with the Cdh1^flox/flox^ genotype and the Villin-Cre genotype in the maternal generation and were also littermates of the Villin-Cre;Cdh1^wt/flox^ mice. To improve understanding, the corresponding context has been modified in the revised manuscript, which can be found in Lines 121-123 and 126.

Again, we thank you for giving us the opportunity to strengthen our manuscript with revisions after your valuable comments and queries. We have worked hard to incorporate your feedback and hope that these revisions persuade you to accept our submission.

Best regards,

Yours sincerely,

Jianping Zhou

Corresponding Author

The First Hospital of China Medical University

E-mail: zjphama@163.com
